# Mild cognitive impairment identification based on motor and cognitive dual-task pooled indices

**DOI:** 10.1371/journal.pone.0287380

**Published:** 2023-08-02

**Authors:** Gianmaria Mancioppi, Erika Rovini, Laura Fiorini, Radia Zeghari, Auriane Gros, Valeria Manera, Philippe Robert, Filippo Cavallo

**Affiliations:** 1 The Department of Industrial Engineering, University of Florence, Florence, Italy; 2 The BioRobotics Institute, Scuola Superiore Sant’Anna, Pontedera, Pisa, Italy; 3 The CoBTeK, Université Côte d’Azur (UCA), Nice, France; 4 Association Innovation Alzheimer, Nice, France; 5 Department of Speech Therapy (Departement d’Orthophonie, DON), Université Côte d’Azur, Nice, France; 6 Centre Hospitalier Universitaire de Nice, Service Clinique Gériatrique du Cerveau et du Mouvement, Centre Mémoire Ressources et Recherche, Université Côte d’Azur, Nice, France; 7 Nice University Hospital, Public Health Department, Côte d’Azur University, Nice, France; Nathan S Kline Institute, UNITED STATES

## Abstract

**Objective:**

This study investigates the possibility of adopting motor and cognitive dual-task (MCDT) approaches to identify subjects with mild cognitive impairment (MCI) and subjective cognitive impairment (SCI).

**Methods:**

The upper and lower motor performances of 44 older adults were assessed using the SensHand and SensFoot wearable system during three MCDTs: forefinger tapping (FTAP), toe-tapping heel pin (TTHP), and walking 10 m (GAIT). We developed five pooled indices (PIs) based on these MCDTs, and we included them, along with demographic data (age) and clinical scores (Frontal Assessment Battery (FAB) scores), in five logistic regression models.

**Results:**

Models which consider cognitively normal adult (CNA) *vs* MCI subjects have accuracies that range from 67% to 78%. The addition of clinical scores stabilised the accuracies, which ranged from 85% to 89%. For models which consider CNA *vs* SCI *vs* MCI subjects, there are great benefits to considering all three regressors (age, FAB score, and PIs); the overall accuracies of the three-class models range between 50% and 59% when just PIs and age are considered, whereas the overall accuracy increases by 18% when all three regressors are utilised.

**Conclusion:**

Logistic regression models that consider MCDT PIs and age have been effective in distinguishing between CNA and MCI subjects. The inclusion of clinical scores increased the models’ accuracy. Particularly high performances in distinguishing among CNA, SCI, and MCI subjects were obtained by the TTHP PI. This study suggests that a broader framework for MCDTs, which should encompass a greater selection of motor tasks, could provide clinicians with new appropriate tools.

## 1 Introduction

The current approach to diagnosing Alzheimer’s disease (AD) and neurocognitive disorders [1] aims at the identification of reliable disease markers [2]. The scientific community is advocating for valid, inexpensive, and non-invasive indicators that may improve the early detection of neurocognitive disorders and the prediction of the trajectory of these disorders [3]. Thanks to the recent widespread availability of digital devices, it may be possible to add new biomarkers to the study of AD [4]. Along with standard exams (neurologic consultation, checks using medical instruments, and neuropsychological testing), new digital biomarkers, with potential prognostic value, could easily be used in clinical and community settings [5, 6]. In this framework, mild neurocognitive disorder (MND) or mild cognitive impairment (MCI), a condition that has a 10-fold risk of progressing to dementia [7], as well as subjective cognitive impairment (SCI), a condition that may precede MCI [8], represent crucial targets for clinical research. The early detection of these conditions and timely interventions have significant benefits for fighting AD and dementia.

The implementation of the motor and cognitive dual-task (MCDT) approach using electronic walkways, optical systems, personal devices, and wearable sensors represents a cutting-edge application in the field of digital biomarkers and the Internet of Medical Things (IoMT) [9]. Motor abnormalities that occur in MCI subjects, which are barely detectable using the naked eye, are emphasised and unmasked using an appropriate brain-stress test / protocols developed to evaluate the functioning of the motor-cognitive interface [10]. Movements require cognitive loads [11], and so do the most common cognitive tasks (*e.g.* counting backwards or naming animals). The intuition behind the MCDT is to combine these two activities, making them compete for the same resources. Cognitive loads consume the subject’s cognitive reserve, subtracting it from the motor-control mechanisms and thus revealing abnormalities [10, 12, 13]. This approach is useful in AD and dementia clinics, as well as for MCI and SCI subjects [9]. Moreover, synthetic indices, called pooled indices (PIs), can be derived from motor parameters extracted during MCDTs. This procedure makes it possible to combine source variables with different scoring ranges into a single summary score [14, 15]. PIs, like other feature reduction techniques, are useful for testing a sizeable number of parameters on a limited number of observations. This is a common situation in clinical studies.

In this study, we present three MCDTs: two tapping tasks (forefinger tapping (FTAP) and toe-tapping heel pin (TTHP)) and the gold standard for MCDT, namely the most common walking task (GAIT). Notably, all the motor parameters were acquired through novel wearable devices for the motion analysis of the upper and lower limbs [16, 17]. Based on the work performed by Keuper et al. [14], we aim to develop new indices to differentiate between cognitively normal adult (CNA) subjects and experimental subjects. Our goal is to enhance the results we obtained in previous studies [18], developing new meaningful scores that, along with standard neurological and neuropsychological exams, could help clinicians to identify at-risk patients.

Thus, we aim (1) to develop MCDT PIs to distinguish MCI subjects from CNA subjects and (2) to distinguish MCI subjects from CNA and SCI subjects. Furthermore, we tried overcoming some of the disadvantages that we observed in the dual-task cost (DTC) calculation.

## 2 Materials and methods

### 2.1 Participants

Seventy older adults were recruited and assessed in this study. Forty-four of them (67%) fulfilled all the requirements and completed the full physical protocol. Seventeen (39%) of these adults had been diagnosed with MND [1], and seventeen (39%) of these adults had SCI. Ten (22%) of these adults were considered CNA subjects. We recruited them at the Memory Centre (CMRR) of Nice University Hospitals (CHU of Nice, France) and the CoBTeK research lab of the Université Cote d’Azur, in the context of the Marco-Sens multi-centric research protocol. Experimental subjects diagnosed with MND (a diagnostic category that stems from the MCI categorisation [19]) and SCI have the following in their records: blood tests, an encephalic MRI, and a neuropsychological assessment, according to the French health authority recommendations. Notably, the MCI subjects were diagnosed using the DSM-5 criteria for MND. On the other hand, the SCI subjects consist of those subjects who complained of cognitive impairments that were not detected by a battery of neuro-psychometric tests. The CNA subjects consisted of those subjects who did not complain of the impairment of cognitive functions and produced cognitively normal results after being assessed using standard neuropsychological screening tests. The exclusion criteria for this study encompass 1) the presence of sensory/motor impairments; 2) the presence of moderate to severe cognitive impairment according to a standard neuropsychological screening test, the Minimental State Examination (MMSE) <24 (adjusted for age and educational level) [20]; 3) the presence of a previous psychiatric disease or other neurological disorders according to clinical interviews; or 4) participation in any cognitive stimulation/training program. Importantly, none of the subjects had problems with understanding questions or counting numbers. We recruited the CNA subjects at the CMRR from among the patients’ caregivers, CMRR personnel, and the CMRR network. The MMSE was employed to confirm that the CNA subjects did not exhibit any signs of cognitive decline. The study was carried out in compliance with the Declaration of Helsinki and obtained approval from the National Ethical Committee (Comité de Protection des Personnes) on 15/04/2019 (N° ID RCB: 2019–A00342–55). All participants received detailed written explanations of the study aims and procedures. They provided their informed written consent before taking part in the study. Notably, this study does not encompasses minors.

### 2.2 Instrumentation

We adopted a wearable system based on microelectromechanical sensors (MEMSs) that was composed of two modules: one for lower limb motor assessment and one for upper limb motor assessment. The first module is a single inertial measurement unit (IMU) integrated into an iNEMO-M1 board (STMicroelectronics, Italy). It consists of a three-axis gyroscope (L3G4200D) for measuring angular velocities, a six-axis geomagnetic module (LSM303DLHC) for acquiring accelerations, and an ARM-based 32-bit microcontroller (STM32F103RE) (STMicroelectronics, Italy). A Velcro strap ensures that the device is on the dorsum of the subjects’ foot, limiting the movement between the foot and the device [16]. On the other hand, the second module consists of four customised IMU-based boards, with one coordination unit included in a bracelet and three more placed on the distal phalanxes of the thumb, index, and middle fingers, within finger thimble packages. The coordination unit communicates with the rings through spiral cables using the Controller Area Network (CAN-bus) standard. Additionally, the bracelet unit synchronises the data exchange between the nodes of the device. The sampling frequency rate is 100 Hz. Bluetooth modules enable the wireless transmission of the acquired data to a remote personal computer. A graphical user interface allows clinicians to store data and to analyse the motion parameters offline [21].

### 2.3 Measures

#### 2.3.1 Neuropsychological assessment

The battery of neuropsychological tests used for the diagnosis of SCI and MCI subjects encompassed the following: 1) the MMSE, which is considered the gold standard for cognitive cortical dementia screening [22]; 2) the Free and Cued Selective Reminding Test (FCSRT), which is used for the assessment of long-term verbal memory; 3) the digit span (direct and inverse), which is used to assess short-term verbal memory and working memory; 4) the verbal fluency (semantic category version), which is used for the assessment of the linguistic repertoire and the mental flexibility; 5) the Trail-Making Test (TMT) form A, which is used for the evaluation of the selective axis of the attention; and finally 6) the TMT form B, which is used to assess the working-memory ability of the subject. Notably, all the subjects (CNA, SCI, and MCI) underwent the Frontal Assessment Battery (FAB), a short screening test that is used to evaluate executive functions [23].

#### 2.3.2 MCDT protocol

Three MCDTs were used in this study: GAIT, FTAP, and TTHP. GAIT represents the gold standard for MCDT assessment. Conversely, FTAP and TTHP, described in [18], are two new MCDT protocols. Importantly, each exercise was performed under both single-task (ST) and dual-task (DT) conditions. The ST condition refers to a motor exercise performed without any cognitive load (CL_0_); the DT condition includes three cognitive load levels (CL_1_, CL_2_, and CL_3_, respectively, counting backwards by ones, by threes, and by sevens) [18]. The experimenter provided instructions about tasks before the subject started the tasks. Furthermore, the experimenter asked the subjects to perform each task at their own pace, without any instruction about task prioritisation (physical task *vs* counting task). Notably, counting backwards was selected as the concurrent cognitive task after a complete literature screening, which indicated that this task is the most commonly used task for MCDT protocols [9].

#### 2.3.3 GAIT

**Fig 1:**
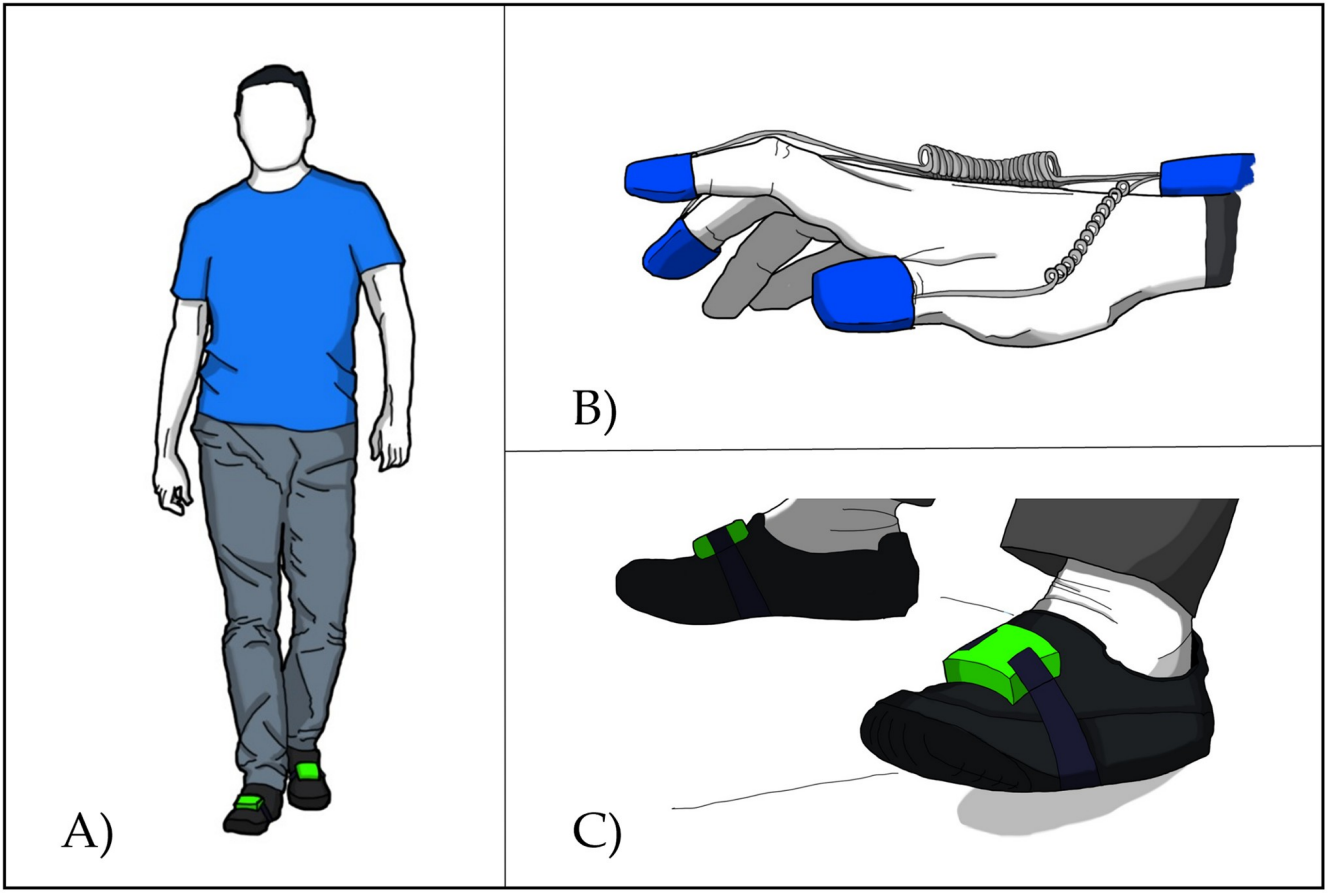
MCDT procedures for identifying cognitive impairment: (A) the GAIT task (walking 10 m in a straight line) performed under four different cognitive loads; (B) the FTAP task (15 s of index-finger tapping) performed under four different cognitive loads; (C) the TTHP task (15 s of toe tapping) performed under four different cognitive loads.

During the GAIT task, a test in which the subject walks 10 m in a straight line, the subject wore the wearable system for lower limb assessment on their dominant foot. The experimenter could objectively assess their performance [24]. The extracted parameters do not take into account the first step and the last step. This makes it possible to avoid acceleration and deceleration phenomena. The subjects were instructed to perform the task at their own pace, without instructions about task prioritisation (see Fig 1A).

#### 2.3.4 FTAP and TTHP

During the FTAP tasks, the subjects had to wear the system for upper limb assessment on their dominant hand. They had to be still, with their hand lying on the table, for 3 s (to acquire the baseline position) and then start tapping their forefinger at their own pace for 15 s (see Fig 1B). Meanwhile, the forearm rests on the desk [24]. On the other hand, during the TTHP task, the subjects had to keep their foot on the ground for 3 s (to acquire the baseline position) and then start tapping their toe for 15 s with their heel resting on the floor (see Fig 1C). The subjects wear the SensFoot device on their dominant foot [24]. Several outcome measures representing the kinematics and kinetics of finger tapping and toe tapping were extracted (see the S1 File). As for GAIT, the subjects were told to perform the task at their own pace, without instructions about task prioritisation (see Fig 1A).

### 2.4 Signal processing and feature extraction

The accelerations and angular velocities acquired by the system were processed offline using MATLAB 2018b^®^ (The MathWorks, Inc., Natick, MA, USA). The signals were filtered and segmented, and motor parameters were extracted following Rovini et al. [17] and Mancioppi et al. [18]. Sixteen features were extracted from the FTAP and TTHP tasks (eight for each task), and the other 16 features came from the GAIT task. The total number of parameters extracted is 32, and these parameters were calculated for each cognitive load. Thus, four datasets were generated (CL_0_, CL_1_, CL_2_, CL_3_). By the end of this process, a total of 128 features had been computed and analysed. The general notation for a feature is *f*_*i*_(*Ex*, CL_*k*_, *s*_*j*_), ∀ *i* ∃{0, …32}, ∀ *k* ∃{0, …3}, ∀ *j* ∃{0, …44}, where *i* is the *i*-th feature computed (as described in Table 1 in the S1 File), *j* is the *j*-th subject performing the task, *Ex* indicates the executed exercise (*Ex* = GAIT, FTAP, TTHP), and *CL*_*k*_ (where *k* = 0, 1, 2, 3) represents the cognitive load level, as described above.

### 2.5 Analyses

#### 2.5.1 Development of a weighted dual-task cost

The DTC represents the difference between a feature extracted while performing the MCDT and the baseline extracted feature. This difference is divided by the baseline and multiplied by 100 (see Eq 1):

$$\begin{array}{c} DTC_{i}\left(Ex,CL_{k},s_{j}\right)=\frac{f_{i}\left(Ex,CL_{k},s_{j}\right)-f_{i}\left(Ex,CL_{0},s_{j}\right)}{f_{i}\left(Ex,CL_{0},s_{j}\right)}\times 100. \end{array}$$ (1)

This technique makes it possible to determine the percentage changes in subjects’ performance on MCDTs. However, we believe that the DTC, as computed so far, does not consider some crucial aspects related to the cognitive side of such protocols. It normalises the subject’s response regarding their motor baseline without acknowledging their cognitive performance. To overcome this limitation, we identify two factors related to the subject’s ability to perform a cognitive task: 1) the cognitive efficiency (the subject’s ability to execute the cognitive task) and 2) the subject’s commitment (how engaged they were). Not considering these data could produce misleading results. For instance, if a subject does not count while performing the task, it could be either due to the fact that the subject is not able to perform the cognitive task (his/her cognitive efficiency is too low) or because the subject is not willing to perform the task (the commitment is too low). This situation can represent a limit case in which the obtained values at *f*_*i*_(*Ex*, *CL*_*k*_, *s*_*j*_) and *f*_*i*_(*Ex*, *CL*_0_, *s*_*j*_) are basically equivalent; therefore, the DTC calculated would be near 0%, and the performance would be considered normal.

We identified the number of correct responses, referred to as *Nc*(*Ex*, *CL*_*k*_, *s*_*j*_), as a proxy for the cognitive efficiency and commitment, combining information about the components. For example, an elevated number of generic responses (both correct and incorrect) tells us that the subject is properly engaged by the task and cooperating but does not tell us about the cognitive efficiency (*i.e.* there is a high number of responses but a low accuracy). Conversely, a high response accuracy tells us that the subject is able to perform the task, but, for example, the subject may stop counting after a couple of subtractions because the task is too difficult and he/she does not want to fail in front of the experimenter or because he/she is not willing to carry out the task (high accuracy but low commitment). On the other side, a high number of correct responses tells us that the subject is properly engaged by the task and that he/she is committed but also tells us something about the subject’s cognitive efficiency.

Thus, we compute an adjusted version of each *f*_*i*_(*Ex*, *CL*_*k*_, *s*_*j*_). It represents the product of *f*_*i*_(*Ex*, *CL*_*k*_, *s*_*j*_) and the respective *Z*-scored *Nc*(*Ex*, *CL*_*k*_, *s*_*j*_), which is referred to as *Z*_*c*_(*Ex*, *CL*_*k*_, *s*_*j*_). Clearly, this calculation is possible only where *k* > 0, and therefore it is not possible at the baseline (see Eq 2):

$$\begin{array}{c} f_{i}{\left(Ex,CL_{k},s_{j}\right)}^{*}=f_{i}\left(Ex,CL_{k},s_{j}\right)\times Z_{c}\left(Ex,CL_{k},s_{j}\right). \end{array}$$ (2)

Notably, we adopted a particular equation (see Eq 3) that forces the distribution to be between 0.01 (to avoid 0 values, which can be difficult to handle) and 1. Note that ‘a’ and ‘b’ refer to the minimum (0.01) and maximum (1) values, respectively:

$$\begin{array}{c} Z_{c}\left(Ex,CL_{k},s_{j}\right)=\left(b-a\right)\frac{Nc\left(Ex,CL_{k},s_{j}\right)-min\left\{Nc\left(Ex,CL_{k},S_{44}^{1}\right)\right\}}{max\left\{Nc\left(Ex,CL_{k},S_{44}^{1}\right)\right\}-min\left\{Nc\left(Ex,CL_{k},S_{44}^{1}\right)\right\}}+a. \end{array}$$ (3)

We used this weighted *f*_*i*_(*Ex*, *CL*_*k*_, *s*_*j*_)* value, instead of the standard one, in the *DTC*_*i*_(*Ex*, *CL*_*k*_, *s*_*j*_) calculation. Hereinafter, we will refer to the *DTC*_*i*_(*Ex*, *CL*_*k*_, *s*_*j*_) values calculated using *f*_*i*_(*Ex*, *CL*_*k*_, *s*_*j*_)*, instead of the general *f*_*i*_(*Ex*, *CL*_*k*_, *s*_*j*_), as *DTC*_*i*_(*Ex*, *CL*_*k*_, *s*_*j*_)*.

#### 2.5.2 Development of pooled indices

**Fig 2:**
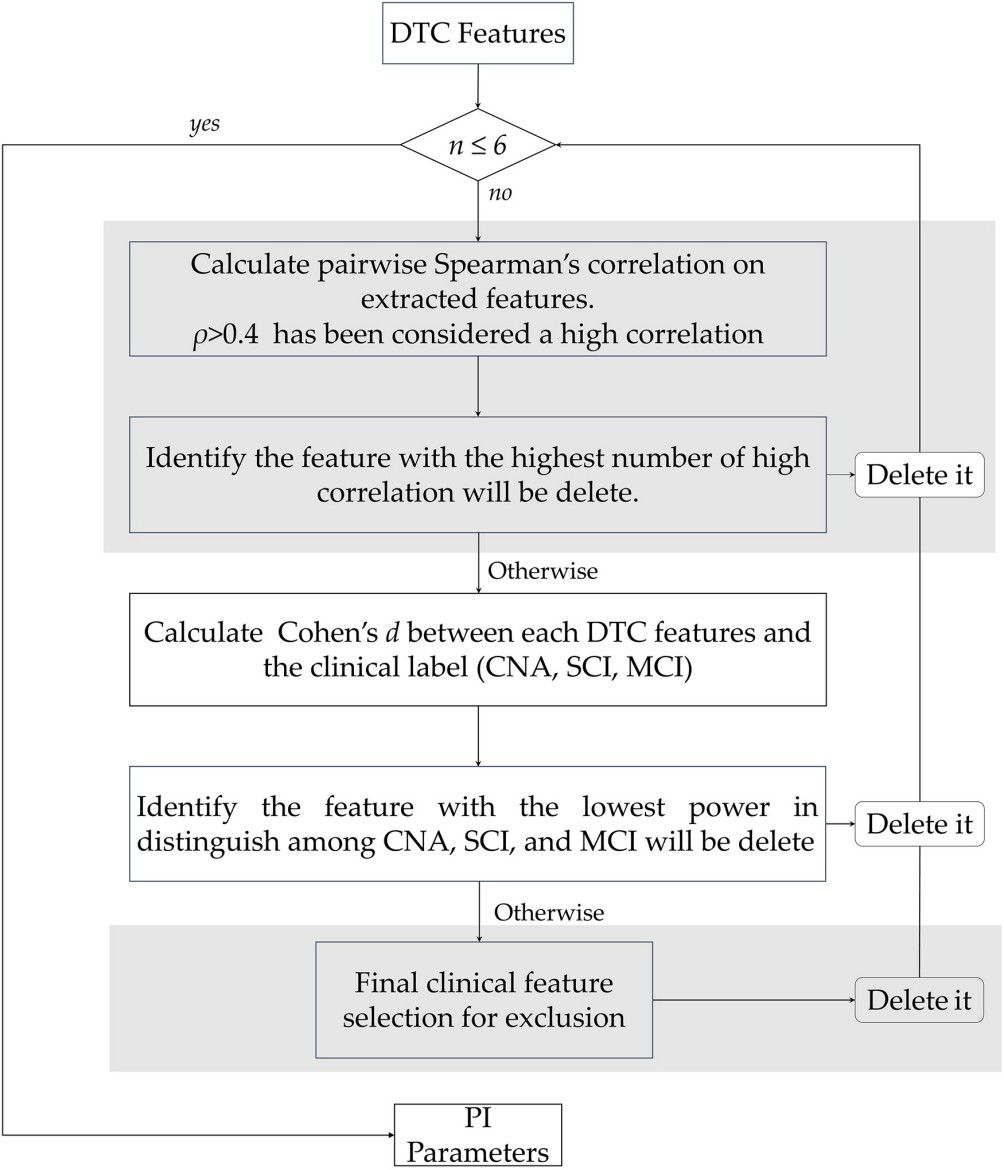
Overview of the development of pooled indices. The criteria we followed throughout the feature selection process are summarised in diamond-shaped blocks. Criterion 1): Is the number of features >6? Criterion 2): Is there a single feature with the highest number of *rho* that are >0.4? Criterion 3): Among the features with the highest number of *rho* that are >0.4, is there a feature with a weaker ability to distinguish among CNA, SCI, and MCI subjects (Cohen’s *d*)? Criterion 4) Among the features screened so far, identify the feature that has less clinical relevance.

To develop a classification model that is able to predict subjects’ diagnostic categories, we decided to adopt a feature reduction approach, which aggregates the DTC* values within each exercise of our MDCT approach in five PIs. In particular, we calculate five PIs for the three aforementioned exercises (FTAP, TTHP, and GAIT), plus 2 additional PIs called TAPPING and TOTAL. TAPPING aggregates the DTCs* values from the tapping tasks (FTAP and TTHP), and TOTAL aggregates the DTCs* values from all three exercises. Hereinafter, we refer to those PIs as *PI*_(*FTAP*)_, *PI*_(*TTHP*)_, *PI*_(*GAIT*)_, *PI*_(*TAPPING*)_, and *PI*_(*TOTAL*)_. Notably, the PIs allow source variables with different scoring ranges to be combined into a single summary score [14]. Importantly, to optimise each PI’s ability to convey information, it is recommended to include in each PI up to six component variables with low pairwise correlations. In this way, it is possible to cover important measurement domains and reduce the variability of the final PI scores [15]. To define up to six features for each PI, we performed the following three steps: (1) we calculated the pairwise Spearman’s correlation among the DTCs* features to select the most unrelated parameters and performed a first feature screening (*i.e.* we looked for FTAP DTCs* with lower correlations to define *PI*_(*FTAP*)_, we looked for TTHP DTCs* with lower correlations to define *PI*_(*HTTP*)_, and so on). A threshold of |*rho*|<0.4 was set [15]. The number of *rho* above the threshold was counted for each feature. The feature that presented the highest number of *rho* above the threshold was deleted. (2) When such a criterion was not applicable (two or more features present the same and highest number of *rho* above the threshold), we deleted the variable that had the lower effect size for the diagnosis; namely, we deleted the feature with the weakest ability to distinguish among the experimental labels (CNA, SCI, and MCI). The effect size was calculated using Cohen’s *d*. (3) Finally, when both the *rho* values and Cohen’s *d* outputs were not able to identify the parameter to be selected, clinical and theoretical considerations were used to determine the more appropriate parameter, as suggested by Keuper et al. [14]. Following this iterative approach, we were able to select up to six DTCs* parameters that then constitute the PIs. Importantly, all the variables were recorded such that higher values indicated a greater level of functioning. This is crucial since the PIs were obtained by first calculating a normalised score for each parameter selected and then averaging those normalised scores in one final PI for each subject [14, 15]. An overview of the PI development process is depicted in Fig 2.

#### 2.5.3 Statistical analysis

We started with preliminary descriptive analyses of the demographic, anthropometric, and clinical measures of the CNA, SCI, and MCI subjects. Differences in gender and educational level within the three groups were assessed using the *χ*^2^ test. On the other hand, since the normality and homoscedasticity assumptions were violated, Kruskal-Wallis tests were adopted to highlight differences among ages and MMSE scores. Post hoc Mann-Whitney tests (corrected using the Bonferroni correction) were employed to identify where the differences occurred. Then, since we needed to solve classification problems with discrete outputs (subjects’ diagnoses: CNA, SCI, and MCI), PI scores were included in multivariate logistic regression models to determine their ability to distinguish subjects from different groups: we used two-class models (CNA *vs* MCI) and three-class models (CNA *vs* SCI *vs* MCI). Different models also included the age and the FAB test score as additional regressors. We included the age as a covariate because MCI (and dementia) is an age-related condition. Therefore, controlling for such a variable could be important. Additionally, we also introduced FAB scores. We aim to add a pure cognitive dimension in our model since the MCDT PIs refer to the motor-cognitive interplay of each subject. The multivariate logistic model results have been validated using a leave-one-out cross-validation method (LOOCV). A summary of the results is presented in the following section. All analyses were performed using MATLAB (version 2018b).

## 3 Results

### 3.1 Socio-demographic and clinical data

Forty-four older adults participated in our study. Among them, 26 were female (59%), and 18 were male (41%). The participants had an overall median age of 69.5 years, with an interquartile range (IQR) of 13.5 years. Seventeen of the participants were diagnosed with MCI. On the other hand, 17 out of 44 were diagnosed with SCI. The remaining ten subjects were considered CNA subjects. The CNA subjects were significantly younger than the SCI and MCI subjects (Kruskal-Wallis p-value <0.05, post-hoc Mann-Whitney (with the Bonferroni correction) p-values <0.01). No significant differences were found between the ages of the SCI and MCI subjects. The neuropsychological measurements, namely the FAB scores, showed statistically significant differences among all the groups (CNA *vs* SCI *vs* MCI). All socio-demographic data and the clinical information are reported in Table 1.

**Table 1:** For each cognition group, the median and IQR values are presented.

Variable	CNA	SCI	MCI	TOTAL	p-value
Number of participants (%)	10 (22%)	17 (39%)	17 (39%)	44	-
Female participants (%)	6 (60%)	7 (41%)	13 (76%)	26 (59%)	0.2
Age, year (IQR)	63 (9)	72 (13.5)	73 (12.75)	69.5 (13.5)	0.02^*a*, *b*^
Education level					0.24
*Primary, n (%)*	0	1 (6%)	3 (18%)	4 (9%)	-
*Secondary, n (%)*	2 (20%)	4 (23%)	6 (35%)	12 (27%)	-
*Superior, n (%)*	8 (80%)	12 (71%)	8 (47%)	28 (64%)	-
FAB (IQR)	18 (1)	17 (1.25)	15 (1.25)	16.5 (2)	<0.01^*a*, *b*, *c*^

The p-values reported for age and the MMSE scores were computed using the Kruskal-Wallis test. *a*, *b*, and *c* indicate significant differences according to the Mann-Whitney post hoc test (with the Bonferroni correction) between pairs of groups. Namely, *a* = difference between CNA and SCI; *b* = difference between CNA and MCI; *c* = difference between SCI and MCI. Differences in gender and educational level were computed using the *χ*^2^ test.

### 3.2 Logistic regression model results

To improve the models’ ability to discern the classes of the subjects (CNA, SCI, and MCI), we consider demographic and clinical variables and MCDT PI values. In particular, we decided to consider the subjects’ age as a covariate due to the age-related nature of MCI and dementia. This also controls for the fact that, as reported in Table 1, the CNA subjects are statistically younger than the SCI and MCI subjects. Furthermore, we added the FAB scores to include a pure neuropsychological dimension. Thus, ten logistic regression models were built using the following combinations of variables: 1) *PI*_(*FTAP*)_ and age; 2) *PI*_(*TTHP*)_ and age; 3) *PI*_(*GAIT*)_ and age; 4) *PI*_(*TAPPING*)_ and age; 5) *PI*_(*TOTAL*)_ and age; 6) *PI*_(*FTAP*)_, age, and FAB score; 7) *PI*_(*TTHP*)_, age, and FAB score; 8) *PI*_(*GAIT*)_, age, and FAB score; 9) *PI*_(*TAPPING*)_, age, and FAB score; and 10) *PI*_(*TOTAL*)_, age, and FAB score. These models were used to distinguish between CNA *vs* MCI subjects and CNA *vs* SCI *vs* MCI subjects. The results are reported in Table 2.

**Table 2:** Cross-validated results of logistic regression models for two-class (CNA *vs* MCI) and three-class (CNA *vs* SCI *vs* MCI) identification.

	CNA *vs* MCI			CNA *vs* SCI *vs* MCI			
Regressors	Sen	Spe	Acc	Rec	Prec	F1-Score	Acc
*PI*_(*FTAP*)_, AGE	82%	63%	70%	50%	46%	47%	50%
*PI*_(*TTHP*)_, AGE	82%	70%	78%	59%	56%	57%	59%
*PI*_(*GAIT*)_, AGE	82%	57%	67%	50%	46%	47%	50%
*PI*_(*TAPPING*)_, AGE	82%	70%	78%	59%	58%	58%	59%
*PI*_(*TOTAL*)_, AGE	76%	60%	70%	55%	54%	54%	55%
*PI*_(*FTAP*)_, AGE, FAB	88%	80%	85%	59%	59%	59%	59%
*PI*_(*HTTP*)_, AGE, FAB	88%	82%	89%	77%	77%	77%	77%
*PI*_(*GAIT*)_, AGE, FAB	88%	80%	85%	66%	66%	66%	66%
*PI*_(*TAPPING*)_, AGE, FAB	88%	80%	85%	70%	71%	71%	70%
*PI*_(*TOTAL*)_, AGE, FAB	88%	80%	85%	68%	69%	68%	68%

Sen = Sensitivity; Spe = Specificity; Acc = Accuracy; Rec = Recall; Pre = Precision. The reported Rec, Pre, and F1-score values represent the weighted averages of these parameters.

**Fig 3:**
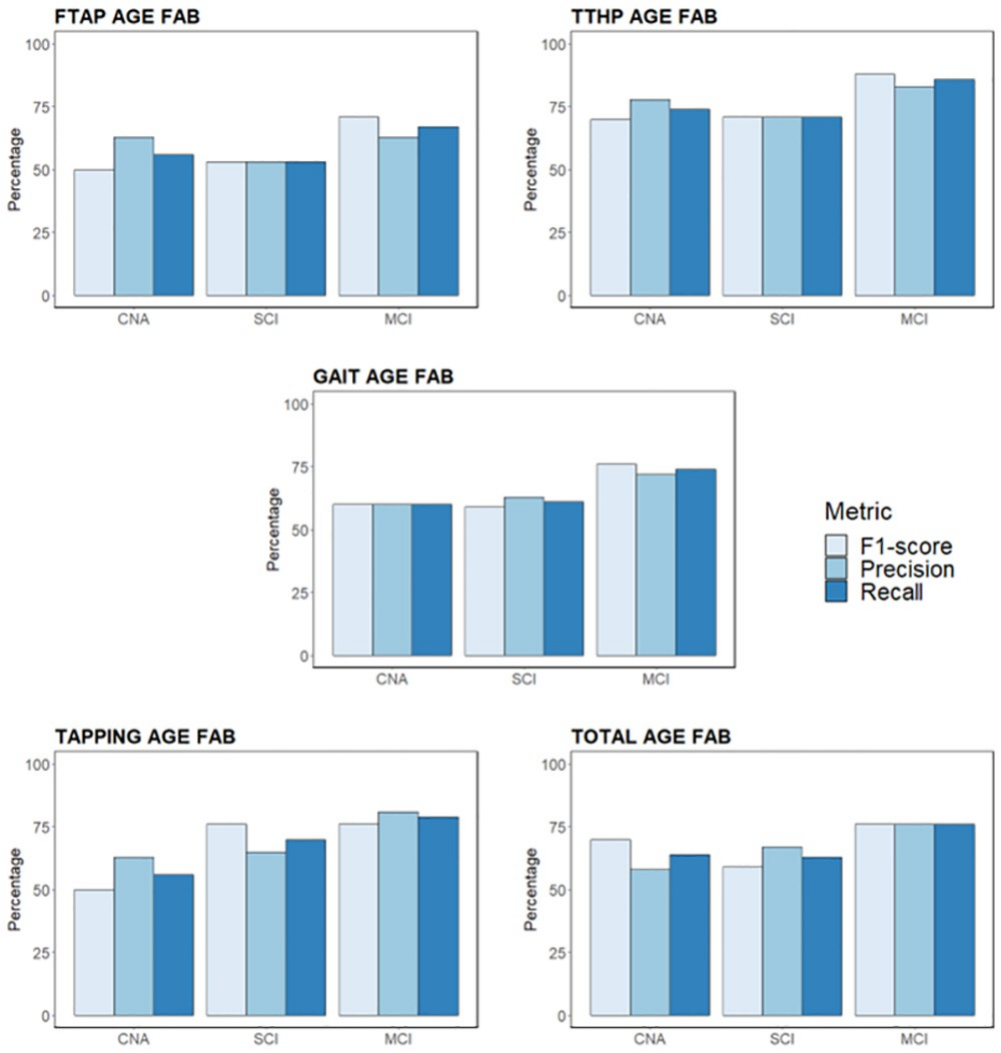
Recall, precision, and F1-score for each experimental group (CNA, SCI, and MCI) for the following models: (A) *PI*_(*FTAP*)_, age, and FAB; (B) *PI*_(*TTHP*)_, age, and FAB; (C) *PI*_(*GAIT*)_, age, and FAB; (D) *PI*_(*TAPPING*)_, age, and FAB; (E) *PI*_(*TOTAL*)_, age, and FAB.

Models that distinguish between CNA *vs* MCI subjects achieve notable outcomes when the PIs and the subjects’ age are used as regressors. The obtained accuracies range from 67% (*PI*_(*GAIT*)_ and age) to 78% (*PI*_(*TTHP*)_, *PI*_(*TAPPING*)_, and age). The addition of FAB scores in the classification model stabilised the accuracies, which, in this case, ranged from the lowest performance of 85% (*PI*_(*FTAP*)_, *PI*_(*GAIT*)_, *PI*_(*TAPPING*)_, *PI*_(*TOTAL*)_, age, and FAB score) to the best result of 89% (*PI*_(*TTHP*)_, age, and FAB score). Notably, the models that encompassed two regressors (PIs and age) obtained high sensitivities, which ranged between 76% and 82%. This reflects the ability of MCDTs and classification models based on PIs and demographic data to identify MCI subjects. The associated specificities do not reach equally good results; they range between 57% and 70%. The FAB scores improved and stabilised the sensitivities, which reached a value of 88%, and specificities (which ranged between 80% and 82%) for all the PIs. On the other hand, models that consider CNA *vs* SCI *vs* MCI subjects benefitted greatly from the adoption of cognitive scores (FAB) in addition to demographic data (age) and PIs. For instance, the overall accuracies of these three-class models range between 50% and 59% if we consider just two regressors (PIs and age). Adopting three regressors (PIs, age, and FAB scores) causes the overall accuracy to increase by 18% (from 59% to 77%). Including a third regressor increases the recall, precision, and F1-score values, as well as the overall accuracy, compared to the two-regressor models (see Table 2). The recalls, precisions, and F1-scores for each group (CNA, SCI, and MCI) and each model built using three regressors (PIs, age, and FAB score) are reported in Fig 3.

## 4 Discussion

This work investigates the possibility of adopting MCDT approaches that use wearable technologies [16, 21] to identify subjects with MCI and SCI. We aimed to enlarge the range of diagnostic solutions available for neurodegenerative diseases that affect the cognitive system. To achieve such a result, we developed ten PIs using a procedure that allows source variables with different scoring ranges to be combined into a single summary score [14]. Then, we utilised these PIs, in combination with demographic data and neuropsychological measurements, to distinguish between CNA and MCI subjects and to distinguish among CNA, SCI, and MCI subjects.

### 4.1 Subject classification performances of the models

The results reported in the previous section (see section 3) showed that models based on PIs were able to distinguish between CNA and MCI subjects, even when just two regressors were used (PIs and age). In particular, referring to this scenario, the best accuracy (78%) was achieved by models that consider subjects’ ages and *PI*_(*TTHP*)_, as well as *PI*_(*TAPPING*)_. Meanwhile, *PI*_(*GAIT*)_, recognised as the MCDT gold standard, had the lowest accuracy level (67%). This is due to the overestimation of the number of MCI subjects. In fact, the model’s specificity rate is 57%. Adding FAB scores to the models causes the sensitivity to increase to 88%, whereas the specificity ranges between 80% and 82%. The *PI*_(*TTHP*)_ model has the highest accuracy (89%), whereas the accuracy of the models with *PI*_(*FTAP*)_, *PI*_(*GAIT*)_, *PI*_(*TAPPING*)_, and *PI*_(*TOTAL*)_ increases to 85%. Conversely, identifying three classes of subjects, namely CNA, SCI, and MCI subjects, represents a more complex task. The models that considered two regressors (PIs and age) achieved a maximum overall accuracy of 59% (*PI*_(*TTHP*)_, *PI*_(*TAPPING*)_, and age). Notably, it seems that the *PI*_(*GAIT*)_ model (the gold standard MCDT that is used to distinguish between CNA and MCI subjects) is not informative enough to distinguish among three classes of subjects. The model that considers *PI*_(*GAIT*)_ and age achieves the lowest overall accuracy level (see Fig 3). In particular, its performance is lower for CNA and SCI subjects, lowering the global model’s efficiency. On the other side, the model that considers *PI*_(*TTHP*)_, age, and the FAB score has the highest overall accuracy levels. This model seems to be considerably better at identifying SCI and MCI subjects, whereas it has a lower efficiency when it comes to CNA subjects (see Fig 3). Using *PI*_(*TAPPING*)_ instead of *PI*_(*TTHP*)_ increases the performance when it comes to identifying CNA subjects. Finally, the use of three regressors makes it easier to distinguish between CNA and MCI subjects or among CNA, SCI, and MCI subjects. In the latter scenario, a pure cognitive measure seems to be necessary to achieve appropriate levels of accuracy. Our study confirms that to distinguish CNA subjects from MCI subjects, the gold standard for MCDT (GAIT) represents an important parameter. The model built using this parameter reaches an accuracy of 85% and a sensitivity of 88% for MCI subjects. However, models built using *PI*_(*FTAP*)_, *PI*_(*TAPPING*)_, and *PI*_(*TOTAL*)_ achieved comparable results, whereas the model built using *PI*_(*TTHP*)_ achieved greater accuracy (+4%) and specificity (+2%) values. The same trend emerged for the models built to distinguish among CNA, SCI, and MCI subjects. GAIT proved to be a helpful tool that was even more useful than FTAP (+6% in the overall recall, precision, and accuracy values), but again, TTHP proved to be the best option in terms of the overall recall, precision, and accuracy for distinguishing among CNA, SCI, and MCI subjects (see Table 2).

Thanks to the combination of demographic, pure cognitive, and MCDT parameters, it is possible to provide clinicians with a brand-new diagnostic support tool for subjects at risk of developing dementia. In particular, it seems that the combination of information about age, scores on tests that measure executive functions, and the performance on MCDTs based on toe tapping led to the best result. Regarding the latter point, it seems that the DTC of parameters related to angular velocities and movement accelerations could be a new crucial frontier in the clinical study of neurodegenerative diseases.

### 4.2 Clinical implications and future directions

In this work, we propose two novel tools for the screening of MCI subjects, namely, FTAP and TTHP. The idea behind this work is that unusual movement could better engage the subjects, but at the same time, the exercises must be simple and must be able to be performed by bedridden subjects, people with reduced mobility, or even during neuroimaging exams. Due to the wearable nature of the system, it is possible to perform FTAP and TTHP tasks everywhere. Neither additional gear (sensorised walkways [13, 25] or optoelectronic systems [26]) nor ample space is required.

Notably, other technologies have been adopted to assess the presence of cognitive decline. These solutions take into account different signals that can be recorded during the evaluation of subjects and rely on personal devices and sensors from consumer-grade devices such as smartphones, smartwatches, tablets, and other wearable systems for physiological tracking. In particular, in [27], the authors investigated the feasibility of utilising multiple smart devices to differentiate between cognitively impaired individuals and healthy individuals. Interestingly, the study adopts an approach that can appropriately handle data quality issues, using device-derived features and demographic data, in order to classify healthy subjects, people with MCI, and subjects with mild AD. However, this study presents some limitations, including, above all, its small sample size. Therefore, the results may not generalise to larger populations or different settings. Finally, the study acknowledges potential ethical concerns regarding the use of automated decision-making tools. On the other hand, in [28], the authors highlight the potential of using the voice as a tool for identifying individuals with dementia, utilising a combination of different verbal, demographic, and clinical features. Digital biomarkers derived from voice analysis are less invasive and less expensive to analyse than traditional biomarkers, can be measured in real time, and can be applied to a wide range of scenarios. However, the study has limitations, including a small sample size and a lack of diversity in the participants, which could impact the generalisability of the findings. Additionally, ethical considerations regarding privacy concerns related to the use of voice data must be addressed.

On the other hand, our work is an attempt to enlarge the framework of the MCDT approach, which is a particular technique used to study the relationship between the motor domain and the cognitive domain. We elaborated a new formula for the DTC calculation, which is described in section 2, with the goal of overcoming the issues we identified during our experimental session, namely, 1) how to evaluate subjects’ efficiency during an MCDT and 2) how to quantify subjects’ commitment during the task. We adopted the procedure described in section 2 to develop synthetic indices that could combine source variables with different scoring ranges into a single summary score. A distinct aspect of our system is that it aims to bridge the gap between clinical expertise and technological advancements by offering a decision support tool that can aid clinicians in making more informed assessments during their patient evaluations. The idea is to provide insights into difficult-to-interpret data by adopting innovative protocols, thereby enhancing the accuracy and efficiency of diagnosis and treatment planning for individuals with cognitive impairment. It builds upon the growing body of literature exploring the potential of technology for supporting clinical decision-making for individuals with cognitive impairment; nevertheless, some limitations are present and there is some room for improvement. First, as mentioned previously, the SCI class makes the classification task more difficult. In particular, the SCI subjects, except for the model that considers *PI*_(*TTHP*)_, ages, and FAB scores, are the subjects that the logistic regression models have the most difficulty identifying. Some of the SCI participants were misclassified as CNA and MCI subjects. We plan to follow these patients longitudinally to see if SCI subjects identified as MCI subjects will show a cognitive decline at the follow-up evaluation and to see if SCI subjects classified as CNA subjects will remain cognitively stable. Moreover, we will schedule new experimental trials to gather more data and increase the size of our sample. The small sample size, particularly for the SCI subgroup, represents the main limitation of this work. Additionally, gathering new data could also help us to overcome limitations regarding age differences among the groups included in this work and could also permit us to stratify the subjects based on the presence of psychiatric symptoms. Emotional disorders could occur in MCI subjects and affect the MCDT performance [29].

In conclusion, synthetic indices gathered from wearable sensors could represent an additional measurement in the framework of digital biomarkers and IoMT. They could also encompass neurophysiological measurements and neuropsychological test scores. Additionally, we plan to assess increasing levels of motor complexity (tapping difficulty). Future directions could also involve studying other clinical populations, such as subjects with Parkinson’s disease (for whom cognitive tasks are often used as distracting tasks to unmask the tremor) [24], stroke patients [30], children suffering from neurodevelopmental disorders [31], people with peripheral neuropathy [32], and amputees [33].

## 5 Conclusions

In conclusion, we aimed to investigate the feasibility of using novel PIs to identify subjects with a higher risk of developing neurodegenerative diseases such as dementia. We developed five PIs to distinguish between CNA and MCI subjects. The use of two regressors (PIs and age) was effective in distinguishing between CNA *vs* MCI subjects. The additional inclusion of FAB scores increased the models’ accuracy. Better performances when it comes to distinguishing between CNA *vs* MCI subjects and CNA *vs* SCI *vs* MCI subjects were obtained above all by the models built using *PI*_(*TTHP*)_. These results suggest that TTHP could be a crucial metric for distinguishing SCI subjects from CNA and MCI subjects. The results obtained in this study suggest that a broader framework for MCDT, which should encompass a greater theoretical framework and selection of tasks, could deepen our knowledge of dementia and provide clinicians with new appropriate tools.

## Supporting information

S1 File The supplementary materials encompass the complete list and descriptions of all the kinematics parameters extracted for the motor and cognitive dual-task protocols. (PDF)

S1 Data (XLSX)
