# Supplementary material for: Mild cognitive impairment identification based on motor and cognitive dual-task pooled indices
Source: PLoS One. 2023 Aug 2;18(8):e0287380. doi: 10.1371/journal.pone.0287380 (PMC10395992; doi:10.1371/journal.pone.0287380)
Supplement: S1 File — (PDF) [file pone.0287380.s001.pdf]

Supplementary Material:
Mild Cognitive Impairment identification based on Motor and Cognitive Dual-Task Pooled Indices

Gianmaria Mancioppi, Erika Rovini, Laura Fiorini, Radia Zeghari, Auriane Gros, Valeria Manera, Philippe Robert, and Filippo Cavallo

The supplementary material encompasses the complete list and the description of all the kinematics parameters extracted for the Motor and Cognitive Dual-Task protocols.

	#	Parameter	Definition
**FTAP & TTHP**			
	1)	Tap	Number of Tapping
	2)	exc	Excursion
	3)	excSD	Excursion SD
	4)	wo	Opening Velocity
	5)	woSD	Opening Velocity SD
	6)	wc	Closing Velocity
	7)	wcSD	Closing Velocity SD
	8)	IAV	Energy Expenditure
**GAIT**			
	1	GT	Gait Time
	2)	GSTRD	Gait Stride
	3)	GVEL	Gait Velocity
	4)	GSTRD-L	Gait Stride Length
	5)	GSTRD-H	Gait Stride Height
	6)	GSTRD-H-SD	Gait Stride Height SD
	7)	GSTRD-T	Gait Stride Time
	8)	GSTRD-T-SD	Gait Stride Time SD
	9)	GSWT	Gait Swing Time
	10)	GSWT-SD	Gait Swing Time SD
	11)	GSTT	Gait Stance Time
	12)	GSTT-SD	Gait Stance Time SD
	13)	GRS	Gait Relative Stance
	14)	GEXC	Gait Excursion
	15)	GEXC-SD	Gait Excursion SD
	16)	GLAT	Gait Latency

Table 1: Complete list and Description of Kinematics Parameters Extracted and adopted in MCDT protocols
